# Leukocyte-to-HDL Ratios for Symptomatic Phenotyping in Heart Failure: Head-to-Head Comparison of Routine Inflammatory Indices and Benchmarking Against NT-proBNP

**DOI:** 10.3390/biomedicines14102222

**Published:** 2026-10-01

**Authors:** Ilija Dragojević, Aleksandra Arsić, Dijana Mirić, Vladan Perić, Dragiša Rašić, Zvezdan Milanović, Anja Kisić, Julijana Rašić, Ivana Belošević, Bojana Kisić

**Affiliations:** 1 Institute of Biochemistry, Faculty of Medicine, University of Priština in Kosovska Mitrovica, 38220 Kosovska Mitrovica, Serbia; miric.dijana@gmail.com (D.M.); bojanabk2002@yahoo.com (B.K.); 2 Department of Medical Biochemistry, Military Hospital, 21000 Novi Sad, Serbia; aleksandrasaskaarsic@gmail.com; 3 Department of Internal Medicine, Faculty of Medicine, University of Priština in Kosovska Mitrovica, 38220 Kosovska Mitrovica, Serbia; vladan.peric@med.pr.ac.rs (V.P.); dragisa.rasic@med.pr.ac.rs (D.R.); 4 Institute of Physiology, Faculty of Medicine, University of Priština in Kosovska Mitrovica, 38220 Kosovska Mitrovica, Serbia; zvezdanmilanovic@yahoo.com; 5 Clinic of Allergy and Immunology, University Clinical Centre of Serbia, 11000 Belgrade, Serbia; anjakisic999@gmail.com; 6 Institute of Pharmacology and Toxicology, Faculty of Medicine, University of Priština in Kosovska Mitrovica, 38220 Kosovska Mitrovica, Serbia; julijana.rasic@med.pr.ac.rs; 7 Clinical Hospital Center, 38220 Kosovska Mitrovica, Serbia; sovrlicivana@yahoo.com

**Keywords:** heart failure, symptomatic severity, NYHA functional class, systemic inflammation, neutrophil-to-HDL ratio, monocyte-to-HDL ratio, NT-proBNP, HDL cholesterol

## Abstract

**Background/Objectives**: Inflammatory indices are increasingly studied in heart failure (HF), but direct comparisons remain limited. We compared eight indices across New York Heart Association (NYHA) classes and assessed the leading indices against N-terminal pro-B-type natriuretic peptide (NT-proBNP). **Methods**: This analysis included 85 patients with established HF. The systemic immune-inflammation index (SII), systemic inflammation response index (SIRI), neutrophil-to-lymphocyte ratio (NLR), neutrophil-to-HDL ratio (NHR), lymphocyte-to-HDL ratio (LHR), monocyte-to-HDL ratio (MHR), platelet-to-HDL ratio (PHR), and uric acid-to-HDL ratio (UHR) were evaluated using group comparisons, Spearman correlations, ordinal logistic regression, and multivariable models for NYHA III/IV versus I/II. Primary multivariable models used a parsimonious adjustment set comprising age, smoking status, and HF duration, while broader covariate adjustment was examined in sensitivity analyses. Internal validation used 1000 bootstrap resamples. **Results**: NHR and PHR differed across NYHA classes after FDR correction. In parsimoniously adjusted ordinal models, SII, SIRI, NLR, NHR, MHR, and PHR were associated with increasing NYHA class after FDR correction. In the NYHA III/IV versus I/II analysis, only NHR (OR per doubling 2.01, 95% CI 1.19–3.39) and MHR (OR 1.96, 95% CI 1.17–3.25) remained significant after FDR correction (q = 0.022 for both). AUCs were 0.688 for the reference model, 0.775 with NHR, 0.777 with MHR, and 0.978 with NT-proBNP. Bootstrap optimism-corrected AUCs were 0.739 for NHR, 0.739 for MHR, and 0.969 for NT-proBNP. Neither ratio improved model fit beyond NT-proBNP. **Conclusions**: NHR and MHR showed the most consistent adjusted associations with NYHA-defined symptomatic severity and remained robust across sensitivity analyses. They should be viewed as compact routine inflammatory-metabolic indices rather than independent biomarkers or replacements for NT-proBNP. External and prospective longitudinal validation is warranted.

## 1. Introduction

Heart failure (HF) encompasses substantial biological and clinical heterogeneity that extends beyond the degree of ventricular dysfunction [1,2,3,4,5]. Alongside cardiac impairment, patients may exhibit varying degrees of neurohormonal activation, vascular dysfunction, metabolic disturbance, and systemic inflammation. Consequently, symptom burden and functional limitation do not necessarily parallel left ventricular ejection fraction (LVEF). The New York Heart Association (NYHA) classification therefore provides a complementary clinical dimension of HF severity [1,2,3]. This distinction is particularly relevant when evaluating routine laboratory markers that may capture the systemic phenotype accompanying symptomatic HF.

Systemic inflammation in HF is accompanied by shifts in circulating leukocyte populations, endothelial dysfunction, oxidative stress, platelet activation, and disturbances in lipid metabolism [4,5,6]. Several composite indices derived from routine laboratory measurements have been proposed as simple surrogates of this systemic response. The systemic immune-inflammation index (SII), systemic inflammation response index (SIRI), and neutrophil-to-lymphocyte ratio (NLR) summarize different relationships among neutrophils, lymphocytes, monocytes, and platelets. A second group of indices combines circulating cell counts with high-density lipoprotein cholesterol (HDL-C), including the neutrophil-to-HDL ratio (NHR), lymphocyte-to-HDL ratio (LHR), monocyte-to-HDL ratio (MHR), and platelet-to-HDL ratio (PHR). These ratios place inflammatory or thrombotic cell populations in relation to circulating HDL-C, while the uric acid-to-HDL ratio (UHR) combines serum uric acid with HDL-C as an index linking metabolic–oxidative burden with the lipoprotein compartment.

Interest in these low-cost indices has grown rapidly, although the available evidence is uneven across markers and clinical settings. SII and SIRI have been studied in relation to HF prevalence, clinical severity, and mortality [7,8,9,10,11], while NLR has a longer literature linking it to impaired functional capacity and adverse outcomes in both chronic and acute HF [12,13]. Evidence for HDL-C-based ratios is more recent. MHR has been associated with prevalent HF and indices of chronic HF severity [11,14], whereas UHR has been linked to both HF prevalence and mortality [15,16]. Studies of NHR have reported associations with prevalent HF, left ventricular dysfunction, and mortality [16,17,18], and mortality-related associations have also been described for LHR [16]. PHR has only recently emerged as a potential HF-related marker [19].

Despite this growing literature, most indices have been examined individually and often in studies centered on HF prevalence, ventricular dysfunction, or prognosis. Direct comparisons among several routinely available indices in patients with HF are uncommon, particularly when symptomatic severity is the primary clinical axis. It therefore remains unclear which indices are most consistently associated with NYHA-defined symptom burden, whether HDL-C-based ratios offer a compact representation of their individual cellular and lipid components, and how they compare with N-terminal pro-B-type natriuretic peptide (NT-proBNP), the established HF biomarker.

The clinical cohort analyzed here has previously been described in a study examining oxidative and nitrosative stress biomarkers in relation to symptomatic and systolic HF severity [20]. The present work addresses a distinct biomarker question. We focused on routine hematological–lipid and inflammatory indices, and healthy controls from the source cohort were not included in the analytical sample. NYHA class served as the principal severity dimension, whereas LVEF was retained as a secondary sensitivity endpoint.

We therefore evaluated eight routinely derived inflammatory and metabolic indices in relation to NYHA-defined symptomatic severity within a common analytical framework. We also examined whether the leading HDL-C-based ratios summarized information from their constituent variables and compared them with NT-proBNP. Analyses based on LVEF were secondary.

## 2. Materials and Methods

### 2.1. Study Design and Analytic Cohort

This study used data from a previously characterized HF cohort [20]. The original study enrolled 85 patients with established chronic HF and 33 healthy reference participants at the Clinic for Internal Diseases, Military Hospital Novi Sad, Serbia, between January 2023 and January 2025. Recruitment was based on predefined eligibility criteria and participant availability rather than consecutive sampling. The HF population included clinically stable inpatients and outpatients.

The current analysis was restricted to the 85 participants with HF; no new participants were enrolled, and no additional samples were collected. Eligibility criteria for the source cohort have been reported in detail previously [20]. In brief, participants were adults aged ≥18 years with written informed consent and clinically established HF supported by transthoracic echocardiography. Patients with acute decompensated HF, acute infection, or another acute inflammatory condition at enrollment were not eligible. Chronic obstructive pulmonary disease and chronic renal failure were also exclusion criteria in the source protocol.

The earlier report from this cohort focused on oxidative and nitrosative stress biomarkers across symptomatic and systolic dimensions of HF severity [20]. The present analysis addressed a distinct biomarker domain: eight indices calculated entirely from routine hematological and biochemical measurements, with symptomatic severity as the primary clinical focus.

The analytic population was restricted to the 85 patients with established HF because the study addressed variation in symptomatic burden rather than case–control discrimination. The 33 healthy participants from the source cohort were therefore excluded from all candidate-index analyses. As the analysis used an existing dataset, no new a priori sample-size calculation was undertaken.

### 2.2. Clinical Assessment and Outcomes

Clinical characterization in the source cohort was based on medical-record review, physician interview, physical examination, and transthoracic echocardiography [20]. Demographic data, smoking status, medical history, comorbidities, HF duration, and treatment information were extracted from the clinical documentation and supplemented by direct patient interview when needed.

The archived analytical dataset recorded rhythm disorders as a broad arrhythmia category and did not permit reliable identification of atrial fibrillation as a separate variable.

HF duration was defined as the interval between the first documented HF diagnosis and study enrollment. When the exact date was not available in the medical record, duration was ascertained from the physician–patient interview and checked against available clinical documentation whenever possible, following the source-study protocol [20].

Functional status was classified according to the NYHA scheme as classes I–IV [1,3], and NYHA class served as the principal severity measure. All four classes were retained for descriptive, correlation, and ordinal regression analyses. For the binary analysis, patients were grouped as NYHA I/II versus NYHA III/IV. The latter was interpreted as greater symptomatic burden rather than “advanced HF,” because NYHA class alone does not define advanced HF. NYHA classification was assigned clinically and independently of NT-proBNP concentration. NYHA class was treated as a clinician-assigned measure of symptomatic and functional burden rather than as an objective measure of global HF severity.

Left ventricular systolic function was assessed by two-dimensional transthoracic echocardiography, with LVEF calculated using the biplane method of disks (modified Simpson’s method) in accordance with chamber-quantification recommendations [20,21]. Patients were classified as having heart failure with preserved ejection fraction (HFpEF; LVEF ≥50%), heart failure with mildly reduced ejection fraction (HFmrEF; LVEF 41–49%), or heart failure with reduced ejection fraction (HFrEF; LVEF ≤40%) [1,3]. Because the study was centered on symptomatic severity, LVEF was treated as a secondary severity axis. Analyses considered both the three-category classification and, where relevant, the binary threshold of LVEF ≤40% versus >40%.

### 2.3. Blood Sampling and Laboratory Measurements

Blood sampling followed the standardized protocol used in the source study [20]. Morning peripheral venous samples were collected after a 12-h overnight fast and processed under uniform preanalytical conditions.

Complete blood counts were obtained on an ADVIA 120 Hematology System (Siemens Healthcare Diagnostics Inc., Tarrytown, NY, USA) using the manufacturer’s reagents and routine analytical procedures. Absolute neutrophil, lymphocyte, monocyte, and platelet counts were recorded in ×10^9^/L and used directly for calculation of the derived indices.

Serum HDL-C and uric acid were measured on a CS-480 automated clinical chemistry analyzer (DIRUI Industrial Co., Ltd., Changchun, China) using original manufacturer reagents. HDL-C was reported in mmol/L and uric acid in µmol/L. Calibration and internal quality control for hematological and biochemical assays followed manufacturer recommendations and routine laboratory procedures.

NT-proBNP was measured by electrochemiluminescence immunoassay on a cobas e 411 analyzer (Roche Diagnostics, Mannheim, Germany) and reported in pg/mL [20]. Two values exceeding the upper reportable concentration of 35,000 pg/mL were set to 35,000 pg/mL for the primary analyses. The NT-proBNP comparison was repeated after exclusion of these observations as a sensitivity check.

All laboratory variables used in the present analysis were obtained at the original enrollment assessment; no new measurements were performed.

### 2.4. Derived Inflammatory and Metabolic Indices

Eight candidate indices were derived from routine hematological and biochemical measurements using established formulas. SII and SIRI were calculated according to definitions previously used in HF populations [8,11], while NLR was calculated from absolute neutrophil and lymphocyte counts [12,13]. NHR, LHR, MHR, and PHR were calculated by dividing the corresponding absolute cell count by HDL-C [14,16,17,18,19], and UHR was calculated as serum uric acid divided by HDL-C [15,16]. The formulas used to calculate the eight derived indices are summarized in Table 1.

Absolute neutrophil, lymphocyte, monocyte, and platelet counts were entered in ×10^9^/L, HDL-C in mmol/L, and serum uric acid in µmol/L. The original laboratory units were retained when calculating the indices. Because these ratios are unit-dependent, their absolute numerical values should be compared across studies only when the same constituent units are used.

### 2.5. Statistical Analysis

Continuous variables are presented as mean ± standard deviation (SD) or median with interquartile range (IQR), as appropriate, and categorical variables as number and percentage. Distributional characteristics were examined before inferential analyses. All primary analyses included the 85 patients in the analytic cohort.

NYHA functional class was designated as the primary severity axis. Differences in the eight derived indices across NYHA classes I–IV were assessed using the Kruskal–Wallis test. Associations with NYHA class, NT-proBNP, and LVEF were evaluated using Spearman’s rank correlation. Benjamini–Hochberg false discovery rate (FDR) correction was applied separately within each predefined family of eight candidate-index tests.

Associations between individual indices and increasing NYHA class were examined in separate proportional-odds ordinal logistic regression models. Given the modest sample size and the number of patients with NYHA III/IV, primary multivariable models used a parsimonious adjustment set comprising age, smoking status, and HF duration. These variables were selected to retain demographic, behavioral, and disease-duration information while limiting model dimensionality. Broader models additionally including sex, body mass index (BMI), and diabetes mellitus were examined as sensitivity analyses to assess the robustness of the primary findings. Odds ratios (ORs) with 95% confidence intervals (CIs) were reported using increments appropriate to the numerical range of each index: per 100 units for SII; per 1 unit for SIRI, NLR, NHR, and LHR; per 0.1 unit for MHR; and per 10 units for PHR and UHR.

For the NYHA III/IV versus I/II analysis, a reference model including age, smoking status, and HF duration was fitted first. Candidate indices were log_2_-transformed so that ORs represented the change in odds of NYHA III/IV associated with a doubling of the respective index. Each index was then added separately to the reference model. Improvement in model fit was assessed using the change in −2 log likelihood and the corresponding likelihood-ratio test. Model discrimination was summarized by the area under the receiver operating characteristic (ROC) curve (AUC), and relative fit by Akaike information criterion (AIC). Broader models additionally including sex, BMI, and diabetes mellitus were examined as sensitivity analyses. AUC estimates were derived from the study sample and represent apparent derivation-sample discrimination.

Because NHR and MHR were the only indices meeting the FDR-adjusted significance threshold in the NYHA III/IV versus I/II analysis, component-decomposition analyses were performed. Ratio models were compared descriptively with corresponding models containing their individual leukocyte and HDL-C components using AUC and AIC, while likelihood-ratio tests assessed improvement over the reference model. These comparisons were intended to assess relative model fit and whether a single ratio could compactly represent its components, not to establish biological independence of the ratios.

NT-proBNP served as the established HF biomarker comparator. NHR and MHR were added separately to the parsimonious reference model plus NT-proBNP to assess whether either ratio improved model fit beyond clinical covariates and NT-proBNP. Comparative results were summarized using AUC and AIC. Internal validation of discrimination was performed using 1000 bootstrap resamples, with optimism estimated from the difference between bootstrap-sample performance and performance of the bootstrap-fitted model when evaluated in the original sample. As sensitivity analyses, the two observations exceeding the upper reportable NT-proBNP concentration of 35,000 pg/mL were excluded, and the comparison was repeated; broader six-covariate models were examined; and the leading NHR and MHR models were additionally adjusted for continuous LVEF in all 85 participants. LVEF-based analyses remained secondary.

All tests were two-sided. For analyses not subject to multiplicity correction, *p* < 0.05 was considered statistically significant; within FDR-adjusted families, significance was defined as q ≤ 0.05. Benjamini–Hochberg FDR correction was applied separately within each prespecified family of eight candidate-index tests; because multiple analytical families were examined, this procedure controlled multiplicity within each family rather than across the entire analytical program. All statistical analyses were performed using IBM SPSS Statistics version 30.0 (IBM Corp., Armonk, NY, USA).

### 2.6. Ethical Considerations

All participants provided written informed consent before enrollment. The source study was conducted in accordance with the Declaration of Helsinki and was approved by the Ethics Committee of the Military Hospital Novi Sad, Serbia (protocol code 284-3; approval date 25 January 2023) [20].

The analysis used data collected within the approved protocol. No additional participants were recruited, biological samples collected, or laboratory measurements performed, and the data were handled in de-identified form.

## 3. Results

### 3.1. HF Cohort

**Figure 1:**
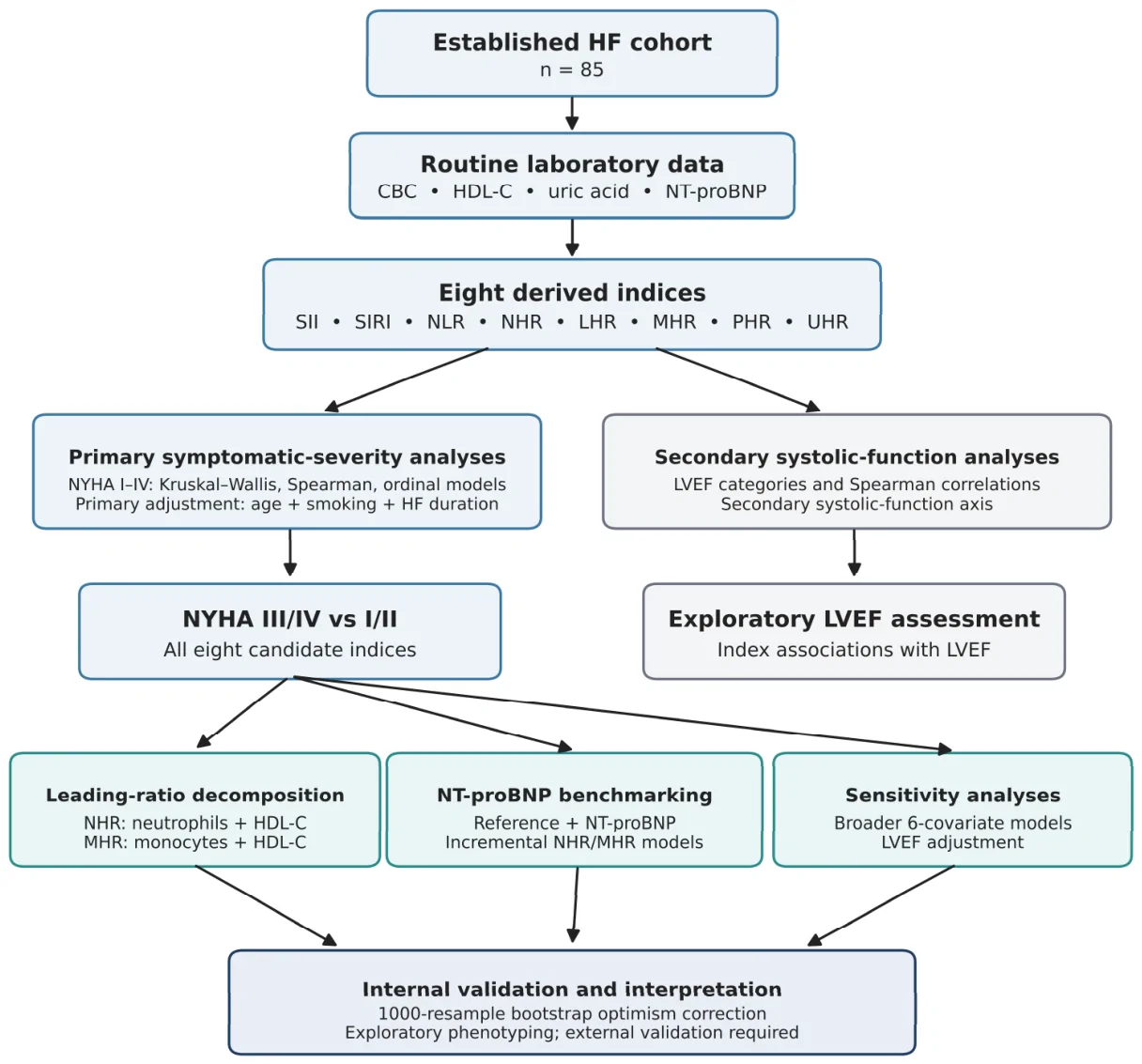
Study design and analytic workflow. The primary analyses examined eight routinely derived inflammatory and meta-bolic indices in relation to NYHA-defined symptomatic severity. All eight candidate indices were compared across NYHA classes and in the NYHA III/IV versus I/II analysis. Primary adjusted analyses used a parsimonious clinical model comprising age, smoking status, and HF duration; broader six-covariate models, bootstrap internal validation, and additional LVEF adjustment were used as sensitivity analyses. NHR and MHR, which met the FDR-adjusted significance threshold in the binary comparison, were subse-quently evaluated by component decomposition and compared with NT-proBNP. LVEF-based analyses were considered secondary. Abbreviations: CBC, complete blood count; FDR, false discovery rate; HDL-C, high-density lipoprotein cholesterol; HF, heart failure; LVEF, left ventricular ejection fraction; MHR, monocyte-to-HDL ratio; NHR, neutrophil-to-HDL ratio; NLR, neutrophil-to-lymphocyte ratio; NT-proBNP, N-terminal pro-B-type natriuretic peptide; NYHA, New York Heart Association functional classi-fication; PHR, platelet-to-HDL ratio; SII, systemic immune-inflammation index; SIRI, systemic inflammation response index; UHR, uric acid-to-HDL ratio.

The analytic population consisted of 85 patients, with all four NYHA functional classes and all three LVEF categories represented. Baseline demographic and clinical characteristics are summarized in Table 2, and the analytic workflow is shown in Figure 1.

NT-proBNP showed a steep monotonic increase across NYHA classes: median (IQR) concentrations were 466 (287–523) pg/mL in NYHA I, 1363 (1052–1927) pg/mL in NYHA II, 4108 (3157–6116) pg/mL in NYHA III, and 20,652 (11,713–23,532) pg/mL in NYHA IV. The patient-level Spearman correlation between NT-proBNP and NYHA class was confirmed at ρ = 0.938 (*p* < 0.001).

### 3.2. NYHA Group Comparisons

The distributions of the eight derived indices across NYHA classes are shown in Table 3. After FDR correction, overall between-class differences remained significant for NHR and PHR. LHR showed a nominal between-class difference (*p* = 0.020) but did not retain statistical significance after FDR adjustment (q = 0.053). None of the remaining indices showed significant overall differences across NYHA classes after multiplicity correction.

### 3.3. Correlations with NYHA, NT-proBNP, and LVEF

**Figure 2:**
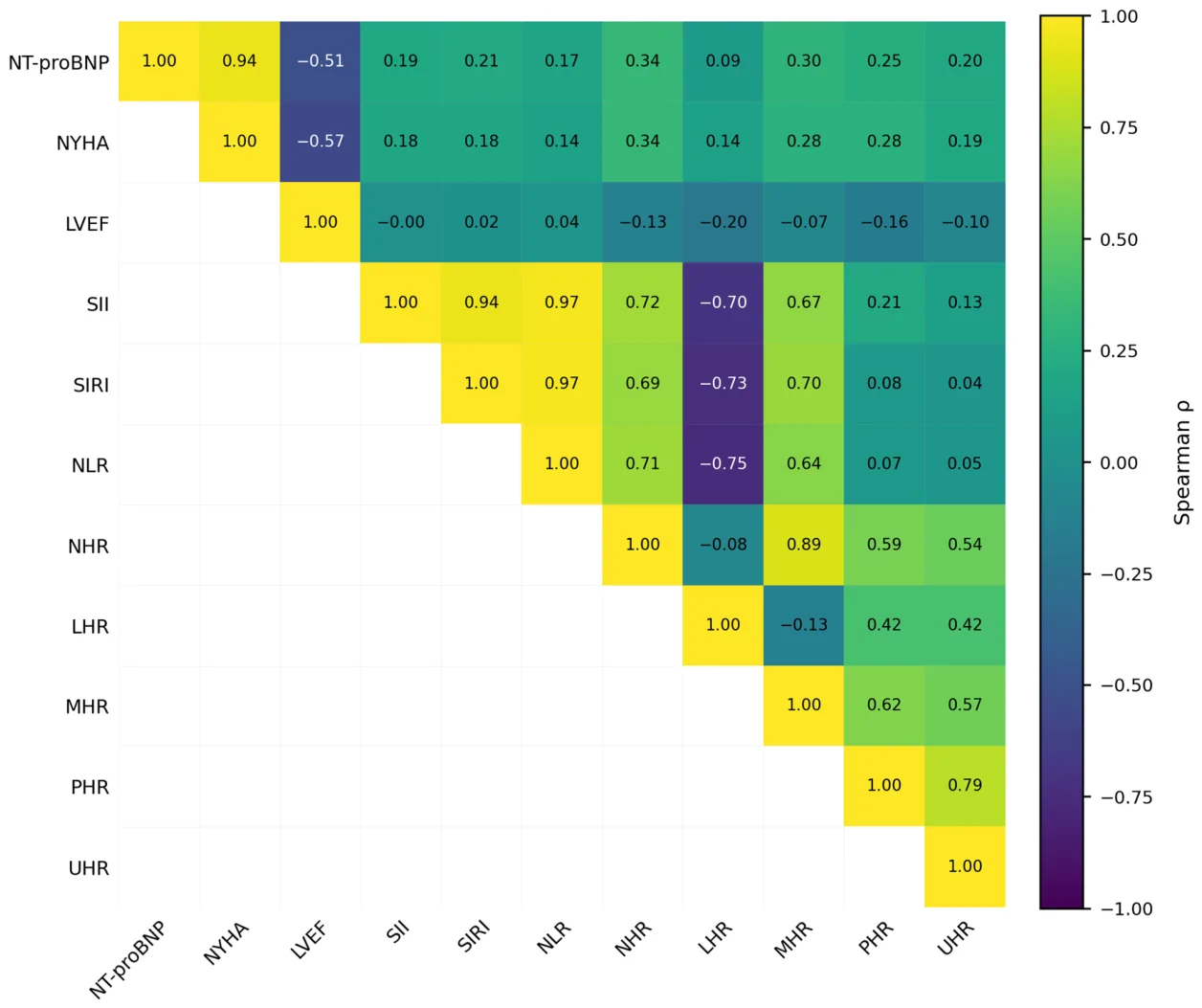
Spearman correlation structure among severity measures and derived inflammatory and metabolic indices. Values shown within cells represent Spearman correlation coefficients (ρ). Only the upper triangular portion of the correlation matrix is displayed to avoid duplication. FDR-adjusted q values for correlations of the candidate indices with NYHA class, NT-proBNP, and LVEF are reported in Table 4. Abbreviations: FDR, false discovery rate; LHR, lymphocyte-to-HDL ratio; LVEF, left ventricular ejection fraction; MHR, monocyte-to-HDL ratio; NHR, neutrophil-to-HDL ratio; NLR, neutrophil-to-lymphocyte ratio; NT-proBNP, N-terminal pro-B-type natriuretic peptide; NYHA, New York Heart Association functional classification; PHR, platelet-to-HDL ratio; SII, systemic immune-inflammation index; SIRI, systemic inflammation response index; UHR, uric acid-to-HDL ratio.

NHR correlated positively with both NYHA class (ρ = 0.341, q = 0.011) and NT-proBNP (ρ = 0.338, q = 0.012) after FDR correction. MHR showed a similar pattern, correlating with NYHA class (ρ = 0.278, q = 0.027) and NT-proBNP (ρ = 0.305, q = 0.018). PHR was also associated with NYHA class (ρ = 0.281, q = 0.027) and showed a weaker association with NT-proBNP at the FDR significance threshold (ρ = 0.255, q = 0.050). None of the eight candidate indices showed an FDR-significant correlation with LVEF. These results are summarized in Table 4 and Figure 2; the full correlation matrix is provided in Supplementary Table S1. Secondary analyses of the candidate indices across LVEF categories are presented in Supplementary Table S2.

### 3.4. Adjusted Ordinal Associations with NYHA Severity

After adjustment for age, smoking status, and HF duration, SII, SIRI, NLR, NHR, MHR, and PHR were significantly associated with increasing NYHA class after FDR correction, whereas LHR and UHR were not (Table 5). Broader six-covariate sensitivity models produced a consistent pattern of results (Supplementary Table S3).

### 3.5. NYHA III/IV Versus I/II Analysis

When all eight candidate indices were evaluated separately against the parsimonious reference model, only NHR and MHR significantly improved model fit after FDR correction. The reference model had an AUC of 0.688; after the addition of NHR or MHR, AUCs increased to 0.775 and 0.777, respectively. NHR was associated with NYHA III/IV with an OR of 2.01 per doubling (95% CI 1.19–3.39), and MHR with an OR of 1.96 (95% CI 1.17–3.25); both had FDR q = 0.022. None of the remaining six indices provided FDR-significant improvement over the reference model (Table 6). The findings were materially unchanged in broader sensitivity models additionally adjusted for sex, BMI, and diabetes mellitus (Supplementary Table S3).

### 3.6. Component Decomposition of NHR and MHR

Component decomposition remained consistent under the parsimonious adjustment strategy. For NHR, the reference + NHR model yielded an AUC of 0.775, compared with 0.773 for reference + neutrophils + HDL-C. For MHR, the reference + MHR model yielded an AUC of 0.777, compared with 0.773 for reference + monocytes + HDL-C. In both cases, the single-ratio models also had lower AIC values than the corresponding two-component models. These findings continue to support NHR and MHR as compact representations of their constituent variables rather than independent biological information (Table 7).

### 3.7. Comparison with NT-proBNP

**Figure 3:**
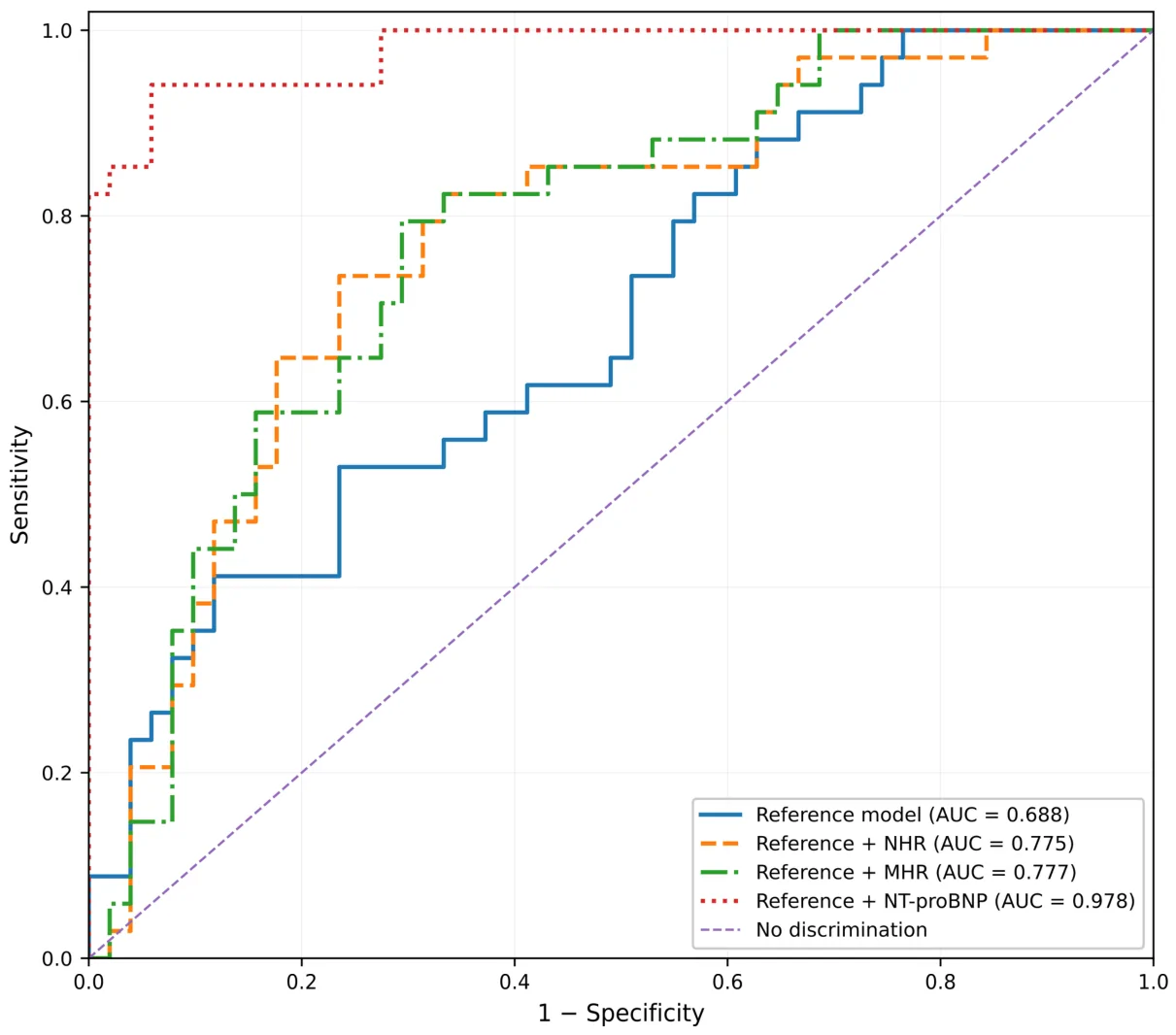
Receiver operating characteristic curves for the reference model and selected biomarker-augmented models for NYHA III/IV versus I/II. Curves are shown for the parsimonious reference model (age, smoking status, and HF duration) alone and after addition of NHR, MHR, or NT-proBNP. AUC values represent apparent derivation-sample discrimination; internally validated values are provided in Supplementary Table S4. The dashed diagonal line represents no discrimination (AUC = 0.50). Abbreviations: AUC, area under the receiver operating characteristic curve; MHR, monocyte-to-HDL ratio; NHR, neutrophil-to-HDL ratio; NT-proBNP, N-terminal pro-B-type natriuretic peptide; NYHA, New York Heart Association functional classification.

NT-proBNP produced a substantially larger improvement in model fit and discrimination than either leading routine ratio. The parsimonious reference + NT-proBNP model achieved an AUC of 0.978, compared with 0.775 for reference + NHR and 0.777 for reference + MHR. Adding NHR or MHR to the reference + NT-proBNP model produced AUCs of 0.978 and 0.981, respectively, and neither addition significantly improved model fit (LR *p* = 0.280 for NHR and *p* = 0.230 for MHR). Thus, although NHR and MHR were associated with NYHA III/IV status, neither ratio improved model fit beyond NT-proBNP (Table 8; Figure 3).

### 3.8. Sensitivity and Internal Validation Analyses

Bootstrap internal validation indicated moderate optimism in the routine-index models. The parsimonious reference model had an apparent AUC of 0.688 and an optimism-corrected AUC of 0.655. The apparent AUCs of 0.775 for the NHR model and 0.777 for the MHR model corresponded to optimism-corrected AUCs of 0.739 for both models. For the NT-proBNP model, the apparent AUC of 0.978 decreased modestly to an optimism-corrected value of 0.969 (Supplementary Table S4).

Exclusion of the two observations above the upper reportable NT-proBNP concentration did not materially alter the benchmarking results; NT-proBNP remained strongly discriminative, and neither NHR nor MHR significantly improved model fit beyond NT-proBNP (Supplementary Table S5).

In a sensitivity analysis additionally adjusting the parsimonious models for continuous LVEF in all 85 participants, NHR remained associated with NYHA III/IV (OR 2.13 per doubling, 95% CI 1.18–3.85, *p* = 0.013), as did MHR (OR 2.19, 95% CI 1.20–4.00, *p* = 0.011). In corresponding ordinal models, both NHR (OR 1.047 per unit, 95% CI 1.012–1.084, *p* = 0.008) and MHR (OR 1.028 per 0.1 unit, 95% CI 1.005–1.051, *p* = 0.016) remained significantly associated with increasing NYHA class. These results are summarized in Supplementary Table S6.

## 4. Discussion

In this cohort of 85 patients with established HF, several routine inflammatory-metabolic indices tracked NYHA-defined symptomatic severity, but NHR and MHR showed the clearest adjusted associations in the NYHA III/IV versus I/II analysis. This pattern was preserved in parsimonious primary models, broader six-covariate sensitivity models, after bootstrap internal validation, and in models additionally adjusted for continuous LVEF in the full cohort. Six indices were associated with increasing NYHA class in the parsimonious ordinal models after FDR correction, whereas only NHR and MHR improved the reference model in the binary analysis. These complementary findings suggest a broader inflammatory-metabolic signal across the NYHA gradient, with a more selective association for NHR and MHR at the NYHA III/IV threshold. This differs from our previous investigation of the same cohort, which focused on oxidative and nitrosative stress markers across symptomatic and systolic HF severity [20].

The parallel findings for NHR and MHR are biologically plausible. Neutrophils and monocytes represent distinct components of innate immune activation, whereas HDL participates in reverse cholesterol transport and is associated with anti-inflammatory, antioxidant, and endothelial-protective functions [4,5,6]. HDL-C concentration, however, does not fully capture HDL functionality. Reduced antioxidant and anti-inflammatory HDL function has been reported in acute HF and impaired antioxidant HDL function in HFpEF [22,23]. Leukocyte-to-HDL ratios may therefore provide simple composite measures of inflammatory cell burden relative to the circulating HDL-C compartment, although they should not be interpreted as direct measures of HDL function.

Previous studies have linked routine inflammatory indices to HF, but most addressed prevalence, ventricular dysfunction, prognosis, or short-term outcomes rather than symptomatic phenotyping. MHR has been associated with prevalent HF and features of chronic HF severity [11,14], whereas NHR has been linked to prevalent HF, left ventricular dysfunction, and mortality [16,17,18]. SII, SIRI, NLR, UHR, LHR, and PHR have also been evaluated in related HF settings [7,8,9,10,12,13,15,16,19]. The present study extends this literature by comparing eight candidate indices within a common adjustment framework and using NYHA-defined symptomatic severity as the primary clinical axis.

Component decomposition is important for interpreting the leading ratios. NHR and MHR are deterministic functions of their constituent variables and should not be regarded as independent biological measurements. Under the parsimonious adjustment strategy, the reference + NHR and reference + neutrophils + HDL-C models again had nearly identical AUCs (0.775 vs. 0.773), as did reference + MHR and reference + monocytes + HDL-C (0.777 vs. 0.773). The ratio models nevertheless represented this information with a single parameter and lower AIC values. NHR and MHR are therefore best viewed as compact composite measures of routine leukocyte and HDL-C information, not as mechanistically superior or independent biomarkers.

The ordinal and binary analyses address related but distinct questions. Ordinal models use information across all four NYHA classes and assess graded associations with symptomatic severity, whereas the NYHA III/IV versus I/II models assess added model fit at a predefined clinical threshold. The broader ordinal signal is therefore compatible with the more selective binary result and should not be interpreted as a discrepancy between analyses.

Comparison with NT-proBNP provides the principal clinical reference point. NT-proBNP showed substantially greater discrimination than either NHR or MHR, and neither ratio significantly improved model fit once NT-proBNP was included. The strong association between NT-proBNP and NYHA class was rechecked at the patient level and was accompanied by a steep monotonic increase in NT-proBNP across individual NYHA classes. Nevertheless, the very high apparent AUC of 0.978 should be interpreted as cohort-specific and potentially optimistic in a modest derivation sample. Bootstrap internal validation reduced the optimism-corrected AUC only modestly to 0.969, but external validation remains essential. These findings do not support leukocyte-to-HDL ratios as replacements for natriuretic peptides or as biomarkers with demonstrated incremental value beyond NT-proBNP; their more plausible role is exploratory inflammatory-metabolic phenotyping using routine laboratory data.

LVEF analyses further emphasize the distinction between symptomatic and systolic severity. None of the eight derived indices showed an FDR-significant association with LVEF in the prespecified secondary analyses. Because ventricular systolic function could nevertheless confound associations with symptomatic burden, the leading NHR and MHR models were additionally adjusted for continuous LVEF in all 85 participants. Both NHR and MHR remained associated with the binary NYHA III/IV versus I/II outcome and with increasing NYHA class in the ordinal sensitivity models. LVEF, however, does not capture the full hemodynamic or myocardial-mechanical phenotype of HF. Global longitudinal strain, detailed diastolic-function measures, and left atrial-left ventricular coupling indices were not acquired in the source protocol and could not be reconstructed retrospectively. These measures should be incorporated into future prospective studies.

Several limitations should be considered. First, this was a modest, single-center, cross-sectional cohort, with only 34 patients classified as NYHA III/IV. Recruitment was based on participant availability rather than consecutive enrollment, which may have introduced selection bias and limits generalizability. Although the primary models were deliberately parsimonious and bootstrap internal validation was performed, some optimism in discrimination estimates may remain, and internal validation does not substitute for external validation. Second, NYHA class is a clinician-assigned and inherently subjective measure of symptomatic and functional burden that may be influenced by age, body habitus, pulmonary disease, physical deconditioning, congestion, comorbidities, and treatment status. Third, residual confounding cannot be excluded. Chronic renal failure was an exclusion criterion in the source cohort, but residual variation in renal function was not incorporated into the models; atrial fibrillation could not be analyzed separately because rhythm disturbances were recorded only as a broad arrhythmia category; and detailed individual-level coding of contemporary HF and cardiometabolic therapies was incomplete, precluding reliable treatment-specific adjustment or stratification. Fourth, although additional adjustment for continuous LVEF did not materially alter the principal NHR and MHR associations, the cohort was not powered for phenotype-specific analyses across HFpEF, HFmrEF, and HFrEF. Moreover, advanced echocardiographic measures, including global longitudinal strain, detailed diastolic-function parameters, and left atrial–left ventricular coupling indices, were not available. Fifth, despite within-family FDR correction, residual multiplicity remains possible across the broader exploratory analytical program. Finally, the cross-sectional design precludes inference regarding directionality, causality, prognosis, monitoring value, or treatment response, and the unit-dependent nature of the derived ratios should be considered when comparing absolute values across studies. Larger prospective multicenter cohorts incorporating objective functional assessment, comprehensive imaging, detailed treatment characterization, and external validation are required.

The study also has strengths. The analytic population was aligned with the specific question of symptomatic variation within established HF; all eight indices were evaluated within a common FDR framework, the leading ratios were compared directly with their components, NT-proBNP provided a clinically relevant benchmark, and the principal NHR/MHR findings were tested across parsimonious, broader six-covariate, bootstrap, and full-cohort LVEF-adjusted sensitivity analyses.

Overall, NHR and MHR emerged as simple composite measures associated with NYHA-defined symptomatic severity. Their role is best considered exploratory systemic phenotyping rather than replacement of established HF biomarkers or routine risk-stratification tools. Prospective multicenter studies with contemporary treatment data, comprehensive echocardiographic phenotyping, and external validation are needed to determine reproducibility and potential clinical utility.

## 5. Conclusions

Among eight routinely derived inflammatory and metabolic indices, NHR and MHR showed the most consistent adjusted associations with NYHA-defined symptomatic severity, and the principal binary associations were preserved across parsimonious, broader six-covariate, and LVEF-adjusted sensitivity analyses. Their discrimination remained similar to that of models containing the corresponding leukocyte and HDL-C components, supporting their interpretation as compact composite measures rather than independent biomarkers. NT-proBNP remained substantially more discriminative, and neither ratio improved model fit beyond NT-proBNP. These findings are hypothesis-generating and require external validation in larger, prospectively characterized HF cohorts with detailed pharmacotherapy and advanced echocardiographic phenotyping.

## Figures and Tables

**Table 1:** Formulas used to calculate the derived inflammatory and metabolic indices.

Index	Formula
SII	platelet count × neutrophil count/lymphocyte count
SIRI	neutrophil count × monocyte count/lymphocyte count
NLR	neutrophil count/lymphocyte count
NHR	neutrophil count/HDL-C
LHR	lymphocyte count/HDL-C
MHR	monocyte count/HDL-C
PHR	platelet count/HDL-C
UHR	serum uric acid/HDL-C

Abbreviations: SII, systemic immune–inflammation index; SIRI, systemic inflammation response index; NLR, neutrophil-to-lymphocyte ratio; NHR, neutrophil-to-HDL ratio; LHR, lymphocyte-to-HDL ratio; MHR, monocyte-to-HDL ratio; PHR, platelet-to-HDL ratio; UHR, uric acid-to-HDL ratio; HDL-C, high-density lipoprotein cholesterol.

**Table 2:** Baseline characteristics of the HF cohort (*n* = 85).

Characteristic	HF Cohort (*n* = 85)
Age, years	63.1 ± 9.7
Male sex, *n* (%)	55 (64.7)
BMI, kg/m^2^	25.0 ± 2.8
Current smoking, *n* (%)	51 (60.0)
Diabetes mellitus, *n* (%)	51 (60.0)
HF duration, years (median, IQR)	5.0 (3.0–9.0)
NYHA class I/II/III/IV, *n* (%)	23 (27.1)/28 (32.9)/19 (22.4)/15 (17.6)
LVEF ≥ 50%/41–49%/≤40%, *n* (%)	20 (23.5)/20 (23.5)/45 (52.9)
NT-proBNP, pg/mL	1511 (551–5547)
ACE inhibitor therapy, *n* (%)	84 (98.8)
Beta-blocker therapy, *n* (%)	46 (54.1)
Diuretic therapy, *n* (%)	47 (55.3)
Cardiotonic therapy, *n* (%)	22 (25.9)

Data are presented as mean ± SD, median (IQR), or *n* (%), as appropriate. Available treatment categories reflect medication information recorded in the source cohort; more granular individual-level coding of contemporary classes such as ARNI, mineralocorticoid receptor antagonists, SGLT2 inhibitors, GLP-1 receptor agonists, and lipid-lowering therapy was not consistently available for retrospective analysis. Abbreviations: BMI, body mass index; HF, heart failure; IQR, interquartile range; LVEF, left ventricular ejection fraction; NT-proBNP, N-terminal pro-B-type natriuretic peptide; NYHA, New York Heart Association functional classification; ACE, angiotensin converting enzyme; SD, standard deviation.

**Table 3:** Derived inflammatory and metabolic indices across NYHA classes.

Index	NYHA I (*n* = 23)	NYHA II (*n* = 28)	NYHA III (*n* = 19)	NYHA IV (*n* = 15)	H	*p*	FDR q
SII	1650.00 (670.84–2327.99)	1791.87 (576.28–4335.72)	1372.50 (560.46–3627.56)	4021.88 (811.76–5141.27)	4.836	0.184	0.246
SIRI	5.27 (0.95–7.15)	5.05 (0.64–11.50)	3.83 (0.74–11.84)	16.00 (1.66–19.33)	3.689	0.297	0.340
NLR	4.11 (1.98–6.91)	4.59 (1.27–11.67)	3.83 (1.34–9.77)	11.33 (1.90–12.86)	2.748	0.432	0.432
NHR	8.51 (6.78–16.77)	18.63 (10.68–31.73)	23.94 (12.60–33.23)	22.22 (16.67–26.16)	12.311	0.006	0.037
LHR	3.33 (2.21–4.38)	4.00 (2.96–6.70)	6.06 (4.00–7.82)	3.40 (1.73–5.33)	9.842	0.020	0.053
MHR	1.59 (0.81–4.00)	2.91 (2.20–4.33)	3.75 (1.83–5.29)	3.14 (2.52–4.60)	7.317	0.062	0.125
PHR	733.33 (611.04–1236.11)	1468.00 (1011.67–1839.13)	1500.00 (970.45–2097.74)	1269.44 (905.00–1959.92)	11.535	0.009	0.037
UHR	955.71 (693.49–1655.00)	1332.34 (910.41–1827.90)	1734.78 (1120.00–1986.01)	1333.33 (901.47–1754.52)	4.831	0.185	0.246

Values are presented as median (IQR). H denotes the Kruskal–Wallis test statistic. Benjamini–Hochberg FDR correction was applied across the eight index-specific comparisons. Abbreviations: FDR, false discovery rate; IQR, interquartile range; LHR, lymphocyte-to-HDL ratio; MHR, monocyte-to-HDL ratio; NHR, neutrophil-to-HDL ratio; NLR, neutrophil-to-lymphocyte ratio; NYHA, New York Heart Association functional classification; PHR, platelet-to-HDL ratio; SII, systemic immune-inflammation index; SIRI, systemic inflammation response index; UHR, uric acid-to-HDL ratio.

**Table 4:** Spearman correlations of derived indices with NYHA class, NT-proBNP, and LVEF.

Index	NYHA ρ	*p*	FDR q	NT-proBNP ρ	*p*	FDR q	LVEF ρ	*p*	FDR q
SII	0.182	0.096	0.134	0.188	0.084	0.112	−0.000	0.996	0.996
SIRI	0.179	0.100	0.134	0.213	0.050	0.101	0.017	0.877	0.996
NLR	0.144	0.188	0.214	0.168	0.125	0.143	0.037	0.735	0.980
NHR	0.341	0.001	0.011	0.338	0.002	0.012	−0.128	0.243	0.647
LHR	0.136	0.214	0.214	0.089	0.418	0.418	−0.200	0.067	0.526
MHR	0.278	0.010	0.027	0.305	0.005	0.018	−0.067	0.545	0.872
PHR	0.281	0.009	0.027	0.255	0.019	0.050	−0.165	0.131	0.526
UHR	0.187	0.086	0.134	0.197	0.070	0.112	−0.098	0.374	0.747

Spearman rank correlation coefficients (ρ) are shown. Benjamini–Hochberg FDR correction was applied separately across the eight candidate indices for each target variable. Statistical significance after FDR correction was defined as q ≤ 0.05. Abbreviations: FDR, false discovery rate; LHR, lymphocyte-to-HDL ratio; LVEF, left ventricular ejection fraction; MHR, monocyte-to-HDL ratio; NHR, neutrophil-to-HDL ratio; NLR, neutrophil-to-lymphocyte ratio; NT-proBNP, N-terminal pro-B-type natriuretic peptide; NYHA, New York Heart Association functional classification; PHR, platelet-to-HDL ratio; SII, systemic immune-inflammation index; SIRI, systemic inflammation response index; UHR, uric acid-to-HDL ratio.

**Table 5:** Parsimoniously adjusted ordinal logistic associations of derived indices with increasing NYHA class.

Index (Scale)	OR	95% CI	*p*	FDR q
SII (per 100)	1.027	1.007–1.048	0.007	0.012
SIRI (per 1)	1.091	1.034–1.151	0.001	0.011
NLR (per 1)	1.117	1.031–1.210	0.007	0.012
NHR (per 1)	1.050	1.017–1.085	0.003	0.012
LHR (per 1)	1.031	0.947–1.122	0.485	0.485
MHR (per 0.1)	1.029	1.007–1.051	0.009	0.012
PHR (per 10)	1.009	1.002–1.015	0.008	0.012
UHR (per 10)	1.004	0.998–1.009	0.176	0.201

Each index was evaluated in a separate proportional-odds model adjusted for age, smoking status, and HF duration. OR scaling is shown in the first column. Benjamini–Hochberg FDR correction was applied across the eight candidate-index models. Broader models additionally adjusted for sex, BMI, and diabetes mellitus are presented in Supplementary Table S3. Abbreviations: CI, confidence interval; FDR, false discovery rate; LHR, lymphocyte-to-HDL ratio; MHR, monocyte-to-HDL ratio; NHR, neutrophil-to-HDL ratio; NLR, neutrophil-to-lymphocyte ratio; NYHA, New York Heart Association functional classification; OR, odds ratio; PHR, platelet-to-HDL ratio; SII, systemic immune-inflammation index; SIRI, systemic inflammation response index; UHR, uric acid-to-HDL ratio.

**Table 6:** Parsimoniously adjusted comparison of eight candidate indices for NYHA III/IV versus I/II.

Index	OR per Doubling	95% CI	LR *p*	FDR q	AUC	AIC
SII	1.22	0.89–1.67	0.200	0.228	0.720	111.00
SIRI	1.20	0.96–1.51	0.107	0.200	0.736	110.04
NLR	1.24	0.90–1.72	0.183	0.228	0.721	110.86
NHR	2.01	1.19–3.39	0.005	0.022	0.775	104.90
LHR	1.18	0.75–1.86	0.464	0.464	0.689	112.10
MHR	1.96	1.17–3.25	0.006	0.022	0.777	104.94
PHR	1.68	0.91–3.12	0.084	0.200	0.721	109.66
UHR	1.54	0.87–2.74	0.125	0.200	0.712	110.28

The reference model included age, smoking status, and HF duration. Each candidate index was log_2_-transformed and added separately to the reference model; ORs therefore represent the change in odds of NYHA III/IV associated with a doubling of the respective index. LR *p*-values compare each expanded model with the reference model; Benjamini–Hochberg FDR correction was applied across the eight LR tests. Higher AUC indicates better discrimination within the study sample, whereas lower AIC indicates better relative model fit. Abbreviations: AIC, Akaike information criterion; AUC, area under the receiver operating characteristic curve; CI, confidence interval; FDR, false discovery rate; HF, heart failure; LHR, lymphocyte-to-HDL ratio; MHR, monocyte-to-HDL ratio; NHR, neutrophil-to-HDL ratio; NLR, neutrophil-to-lymphocyte ratio; NYHA, New York Heart Association functional classification; OR, odds ratio; PHR, platelet-to-HDL ratio; SII, systemic immune-inflammation index; SIRI, systemic inflammation response index; UHR, uric acid-to-HDL ratio.

**Table 7:** Component decomposition of the leading HDL-C–leukocyte ratio models.

Model	AUC	AIC	LR *p* vs. Reference	Biomarker OR(s) per Doubling
Reference model	0.688	110.64	—	—
Reference + neutrophils	0.737	109.39	0.071	Neutrophils 1.83 (0.93–3.60)
Reference + HDL-C	0.743	107.81	0.028	HDL-C 0.44 (0.20–0.94)
Reference + neutrophils + HDL-C	0.773	106.72	0.019	Neutrophils 1.82 (0.91–3.63); HDL-C 0.44 (0.20–0.96)
Reference + NHR	0.775	104.90	0.005	NHR 2.01 (1.19–3.39)
Reference + monocytes	0.747	108.90	0.053	Monocytes 1.90 (0.96–3.79)
Reference + monocytes + HDL-C	0.773	106.85	0.020	Monocytes 1.79 (0.89–3.61); HDL-C 0.46 (0.21–1.01)
Reference + MHR	0.777	104.94	0.006	MHR 1.96 (1.17–3.25)

All models include the parsimonious reference covariates age, smoking status, and HF duration. LR *p*-values compare each expanded model with the reference model. ORs are reported per doubling of the indicated biomarker or component. AUC values are study-sample estimates. Because NHR and MHR are deterministic functions of their constituent variables, comparisons between ratio and component models are descriptive and intended to assess relative model fit and compact representation rather than statistical independence. Abbreviations: AIC, Akaike information criterion; AUC, area under the receiver operating characteristic curve; HDL-C, high-density lipoprotein cholesterol; LR, likelihood ratio; MHR, monocyte-to-HDL ratio; NHR, neutrophil-to-HDL ratio; OR, odds ratio.

**Table 8:** Comparison of the leading routine indices with NT-proBNP for NYHA III/IV versus I/II.

Model	AUC	AIC	LR *p*
Reference model	0.688	110.64	—
Reference + NHR	0.775	104.90	0.005
Reference + MHR	0.777	104.94	0.006
Reference + NT-proBNP	0.978	40.31	<0.001
Reference + NT-proBNP + NHR	0.978	41.15	0.280
Reference + NT-proBNP + MHR	0.981	40.88	0.230

The reference model includes age, smoking status, and HF duration. For reference + NHR, reference + MHR, and reference + NT-proBNP, LR *p*-values compare the expanded model with the reference model. For reference + NT-proBNP + NHR and reference + NT-proBNP + MHR, LR *p*-values compare the expanded model with the reference + NT-proBNP model. AUC values are apparent derivation-sample estimates; bootstrap optimism-corrected values are reported in Supplementary Table S4. Abbreviations: AIC, Akaike information criterion; AUC, area under the receiver operating characteristic curve; LR, likelihood ratio; MHR, monocyte-to-HDL ratio; NHR, neutrophil-to-HDL ratio; NT-proBNP, N-terminal pro-B-type natriuretic peptide; NYHA, New York Heart Association functional classification.

## Data Availability

The data presented in this study are available on request from the corresponding author due to hospital policies.

## Supplementary Materials

The following supporting information can be downloaded at https://www.mdpi.com/article/10.3390/biomedicines14102222/s1. Table S1, Full Spearman correlation matrix; Table S2, Secondary LVEF analyses; Table S3, Broader six-covariate sensitivity analyses; Table S4, Bootstrap internal validation of model discrimination; Table S5, Sensitivity analysis for NT-proBNP values above the upper reportable concentration; Table S6, Sensitivity analyses additionally adjusted for continuous LVEF.

## Abbreviations

The following abbreviations are used in this manuscript:

AIC	Akaike information criterion
AUC	area under the receiver operating characteristic curve
BMI	body mass index
CBC	complete blood count
CI	confidence interval
FDR	false discovery rate
HDL-C	high-density lipoprotein cholesterol
HF	heart failure
HFmrEF	heart failure with mildly reduced ejection fraction
HFpEF	heart failure with preserved ejection fraction
HFrEF	heart failure with reduced ejection fraction
IQR	interquartile range
LHR	lymphocyte-to-HDL ratio
LR	likelihood ratio
LVEF	left ventricular ejection fraction
MHR	monocyte-to-HDL ratio
NHR	neutrophil-to-HDL ratio
NLR	neutrophil-to-lymphocyte ratio
NT-proBNP	N-terminal pro-B-type natriuretic peptide
NYHA	New York Heart Association
OR	odds ratio
PHR	platelet-to-HDL ratio
ROC	receiver operating characteristic
SD	standard deviation
SII	systemic immune-inflammation index
SIRI	systemic inflammation response index
UHR	uric acid-to-HDL ratio
