# Supplementary material for: Leukocyte-to-HDL Ratios for Symptomatic Phenotyping in Heart Failure: Head-to-Head Comparison of Routine Inflammatory Indices and Benchmarking Against NT-proBNP
Source: Biomedicines. 2026 Oct 1;14(10):2222. doi: 10.3390/biomedicines14102222 (PMC13701271; doi:10.3390/biomedicines14102222)
Supplement: Supplementary file 1 [file biomedicines-14-02222-s001.zip › biomedicines-4589627-supplementary.pdf]

Supplementary Tables

Table S1. Full Spearman correlation matrix.

Variable	NT-proBNP	NYHA	LVEF	SII	SIRI	NLR	NHR	LHR	MHR	PHR	UHR
NT-proBNP	1.00	0.94 (<0.001)	-0.51 (<0.001)	0.19 (0.084)	0.21 (0.050)	0.17 (0.125)	0.34 (0.002)	0.09 (0.418)	0.30 (0.005)	0.25 (0.019)	0.20 (0.070)
NYHA	0.94 (<0.001)	1.00	-0.57 (<0.001)	0.18 (0.096)	0.18 (0.100)	0.14 (0.188)	0.34 (0.001)	0.14 (0.214)	0.28 (0.010)	0.28 (0.009)	0.19 (0.086)
LVEF	-0.51 (<0.001)	-0.57 (<0.001)	1.00	-0.00 (0.996)	0.02 (0.877)	0.04 (0.735)	-0.13 (0.243)	-0.20 (0.067)	-0.07 (0.545)	-0.16 (0.131)	-0.10 (0.374)
SII	0.19 (0.084)	0.18 (0.096)	-0.00 (0.996)	1.00	0.94 (<0.001)	0.97 (<0.001)	0.72 (<0.001)	-0.70 (<0.001)	0.67 (<0.001)	0.21 (0.053)	0.13 (0.242)
SIRI	0.21 (0.050)	0.18 (0.100)	0.02 (0.877)	0.94 (<0.001)	1.00	0.97 (<0.001)	0.69 (<0.001)	-0.73 (<0.001)	0.70 (<0.001)	0.08 (0.493)	0.04 (0.709)
NLR	0.17 (0.125)	0.14 (0.188)	0.04 (0.735)	0.97 (<0.001)	0.97 (<0.001)	1.00	0.71 (<0.001)	-0.75 (<0.001)	0.64 (<0.001)	0.07 (0.521)	0.05 (0.677)
NHR	0.34 (0.002)	0.34 (0.001)	-0.13 (0.243)	0.72 (<0.001)	0.69 (<0.001)	0.71 (<0.001)	1.00	-0.08 (0.471)	0.89 (<0.001)	0.59 (<0.001)	0.54 (<0.001)
LHR	0.09 (0.418)	0.14 (0.214)	-0.20 (0.067)	-0.70 (<0.001)	-0.73 (<0.001)	-0.75 (<0.001)	-0.08 (0.471)	1.00	-0.13 (0.253)	0.42 (<0.001)	0.42 (<0.001)
MHR	0.30 (0.005)	0.28 (0.010)	-0.07 (0.545)	0.67 (<0.001)	0.70 (<0.001)	0.64 (<0.001)	0.89 (<0.001)	-0.13 (0.253)	1.00	0.62 (<0.001)	0.57 (<0.001)
PHR	0.25 (0.019)	0.28 (0.009)	-0.16 (0.131)	0.21 (0.053)	0.08 (0.493)	0.07 (0.521)	0.59 (<0.001)	0.42 (<0.001)	0.62 (<0.001)	1.00	0.79 (<0.001)
UHR	0.20 (0.070)	0.19 (0.086)	-0.10 (0.374)	0.13 (0.242)	0.04 (0.709)	0.05 (0.677)	0.54 (<0.001)	0.42 (<0.001)	0.57 (<0.001)	0.79 (<0.001)	1.00

Values are Spearman correlation coefficients (ρ), with nominal two-sided p values in parentheses. This table is provided to show the correlation structure among the principal clinical variables and the eight derived indices. FDR-adjusted q values for correlations of the candidate indices with NYHA class, NT-proBNP, and LVEF are reported in Table 4. Abbreviations: HF, heart failure; LHR, lymphocyte-to-HDL ratio; LVEF, left ventricular ejection fraction; MHR, monocyte-to-HDL ratio; NHR, neutrophil-to-HDL ratio; NLR, neutrophil-to-lymphocyte ratio; NT-proBNP, N-terminal pro-B-type natriuretic peptide; NYHA, New York Heart Association functional classification; PHR, platelet-to-HDL ratio; SII, systemic immune-inflammation index; SIRI, systemic inflammation response index; UHR, uric acid-to-HDL ratio.

Table S2. Secondary LVEF analyses.

Index	Kruskal–Wallis H	p	FDR q	Adjusted ordinal OR	Ordinal p	Ordinal FDR q
SII	0.633	0.729	0.866	1.017	0.145	0.217
SIRI	0.296	0.862	0.866	1.051	0.107	0.217
NLR	0.289	0.866	0.866	1.072	0.133	0.217
NHR	7.073	0.029	0.116	1.040	0.040	0.217
LHR	3.807	0.149	0.298	1.089	0.163	0.217
MHR	2.778	0.249	0.399	1.013	0.290	0.331
PHR	5.824	0.054	0.145	1.007	0.068	0.217
UHR	8.097	0.017	0.116	1.003	0.410	0.410

LVEF was analyzed as a secondary sensitivity endpoint. Kruskal–Wallis tests compared the eight candidate indices across LVEF categories (≥50%, 41–49%, and ≤40%). Adjusted ordinal models used worsening LVEF category as the ordered outcome and included age, sex, BMI, smoking status, diabetes mellitus, and HF duration. Benjamini–Hochberg FDR correction was applied separately across the eight indices within each LVEF analysis family. No association remained significant after FDR correction. Abbreviations: FDR, false discovery rate; HF, heart failure; LHR, lymphocyte-to-HDL ratio; LVEF, left ventricular ejection fraction; MHR, monocyte-to-HDL ratio; NHR, neutrophil-to-HDL ratio; NLR, neutrophil-to-lymphocyte ratio; OR, odds ratio; PHR, platelet-to-HDL ratio; SII, systemic immune-inflammation index; SIRI, systemic inflammation response index; UHR, uric acid-to-HDL ratio.

Table S3. Broader six-covariate sensitivity analyses.

Panel A. Ordinal NYHA models with broader six-covariate adjustment.

Index (scale)	OR	95% CI	p	FDR q	PO test p
SII (per 100)	1.028	1.007–1.050	0.009	0.018	0.371
SIRI (per 1)	1.097	1.036–1.162	0.002	0.012	0.527
NLR (per 1)	1.123	1.032–1.223	0.007	0.018	0.526
NHR (per 1)	1.048	1.013–1.083	0.006	0.018	0.130
LHR (per 1)	1.032	0.943–1.131	0.490	0.490	0.253
MHR (per 0.1)	1.027	1.005–1.050	0.016	0.022	0.272
PHR (per 10)	1.009	1.002–1.015	0.014	0.022	0.173
UHR (per 10)	1.003	0.997–1.009	0.286	0.327	0.396

Panel B. Binary NYHA III/IV versus I/II models with broader six-covariate adjustment.

Index	OR per doubling	95% CI	LR p	FDR q	AUC	AIC
SII	1.27	0.91–1.77	0.153	0.175	0.749	114.35
SIRI	1.24	0.97–1.58	0.072	0.175	0.758	113.16
NLR	1.30	0.92–1.83	0.131	0.175	0.750	114.11
NHR	2.01	1.18–3.42	0.007	0.026	0.795	109.00
LHR	1.13	0.69–1.85	0.621	0.621	0.731	116.15
MHR	2.01	1.18–3.43	0.006	0.026	0.788	108.72
PHR	1.66	0.86–3.18	0.116	0.175	0.741	113.92
UHR	1.53	0.85–2.78	0.143	0.175	0.742	114.25

The broader reference models included age, sex, BMI, smoking status, diabetes mellitus, and HF duration. These analyses were examined to assess whether the principal NHR/MHR findings were robust to additional covariate adjustment beyond the parsimonious primary models. Benjamini–Hochberg FDR correction was applied within each eight-index analysis family.

Table S4. Bootstrap internal validation of model discrimination.

Model	Apparent AUC	Estimated optimism	Optimism-corrected AUC	Valid resamples
Parsimonious reference model	0.688	0.033	0.655	1000
Reference + NHR	0.775	0.037	0.739	1000
Reference + MHR	0.777	0.038	0.739	1000

Reference + NT-proBNP	0.978	0.009	0.969	948

Internal validation used 1000 bootstrap resamples. Optimism was estimated as the mean difference between AUC in the bootstrap sample and AUC obtained when the bootstrap-fitted model was evaluated in the original sample. For the NT-proBNP model, 948 unpenalized bootstrap fits were valid; some resamples exhibited separation or non-convergence. These results quantify internal optimism but do not substitute for external validation.

Table S5. Sensitivity analysis for NT-proBNP values above the upper reportable concentration.

**Analysis**	**Primary handling**	**Sensitivity approach**	**Result**
NT-proBNP comparison	Two NT-proBNP values reported >35,000 pg/mL were entered as 35,000 pg/mL	Both observations were excluded and the principal benchmarking models were repeated (n = 83)	Reference + NT-proBNP AUC was 0.977. Addition of NHR did not increase AUC (0.977; LR p = 0.280), and addition of MHR yielded an AUC of 0.980 (LR p = 0.230). Neither ratio significantly improved model fit. The principal conclusion was unchanged.

This sensitivity analysis examined whether handling of NT-proBNP concentrations above the analyzer's reportable range influenced the comparison. Models used the parsimonious reference covariates (age, smoking status, and HF duration). AUC values are study-sample estimates. Abbreviations: AUC, area under the receiver operating characteristic curve; LR, likelihood ratio; MHR, monocyte-to-HDL ratio; NHR, neutrophil-to-HDL ratio; NT-proBNP, N-terminal pro-B-type natriuretic peptide.

Table S6. Sensitivity analyses additionally adjusted for continuous LVEF.

Panel A. Binary NYHA III/IV versus I/II models additionally adjusted for continuous LVEF.

Index	OR per doubling	95% CI	p
NHR	2.13	1.18–3.85	0.013
MHR	2.19	1.20–4.00	0.011

Panel B. Ordinal NYHA models additionally adjusted for continuous LVEF.

Index	Adjusted ordinal OR	95% CI	p
NHR (per 1 unit)	1.047	1.012–1.084	0.008
MHR (per 0.1 unit)	1.028	1.005–1.051	0.016

All models included age, smoking status, and HF duration plus continuous LVEF and used the full HF cohort (n = 85). ORs for the binary models are reported per doubling of NHR or MHR. Ordinal ORs retain the scaling used in the primary ordinal analyses.
